# Supplementary material for: Analysis of PPARγ Signaling Activity in Psoriasis
Source: Int J Mol Sci. 2021 Aug 10;22(16):8603. doi: 10.3390/ijms22168603 (PMC8395241; doi:10.3390/ijms22168603)
Supplement: Supplementary file 1 [file ijms-22-08603-s001.zip › Supplemental materials_Analysis of PPARg signaling activity in psoriasis/Pathway models/Models images and html files/PPARG expressed in cells assotiated with psoriasis/225509.html]

macrophage


# CellType macrophage

|  |  |
| --- | --- |
| URN | urn:agi-ncimcelltype:C1550652 |
| Total Entities | 92 |
| Connectivity | 15404 |
| Name | macrophage |

---

|  |  |
| --- | --- |
| ChildConcepts | macrophage function |
|  | macrophage interaction |
|  | macrophage adhesion |
|  | macrophage differentiation |
|  | macrophage chemotaxis |
|  | macrophage migration |
|  | macrophage apoptosis |
|  | CD169+ macrophage |
|  | lipophage |
|  | M1 macrophage |
|  | M2 macrophage |
|  | muciphage |
|  | histiocyte |
|  | adipose macrophage |
|  | bone marrow macrophage |
|  | central nervous system macrophage |
|  | choroid plexus macrophage |
|  | melanophage |
|  | meningeal macrophage |
|  | perivascular macrophage |
|  | pleural macrophage |
|  | thymic macrophage |
|  | tumor infiltrating macrophage |
|  | nurse cell |
|  | monocyte-derived nurse-like cell |
|  | Kuppfer cell |
|  | peritoneal macrophage |
|  | alveolar macrophage |
|  | macrophage infiltration |
|  | macrophage polarity |
|  | macrophage phenotype |
|  | macrophage priming |
|  | macrophage population |
|  | macrophage death |
|  | macrophage proliferation |
|  | macrophage formation |
|  | macrophage survival |
|  | macrophage recognition |
|  | macrophage aggregation |
|  | macrophage development |
|  | macrophage extravasation |
|  | macrophage sequestration |
|  | macrophage transdifferentiation |
|  | macrophage phagocytosis |
|  | macrophage motion |
|  | macrophage motility |
|  | macrophage invasion |
|  | macrophage homing |
|  | macrophage growth |
|  | macrophage cytotoxicity |
|  | macrophage damage |
|  | macrophage localization |
|  | macrophage phagocyte activity |
|  | macrophage senescence |
|  | macrophage mediated cytotoxicity |
|  | macrophage autophagy |
|  | macrophage repertoire |
|  | macrophage glial cell function |
|  | macrophage ratio |
|  | macrophage homeostasis |
|  | macrophage spreading |
|  | macrophage transendothelial migration |
|  | macrophage division |
|  | macrophage enlargement |
|  | macrophage fate |
|  | macrophage size |
|  | macrophage renewal |
|  | macrophage destruction |
|  | macrophage clonal anergy |
|  | macrophage communication |
|  | macrophage stress |
|  | macrophage regeneration |
|  | macrophage distribution |
|  | macrophage fate commitment |
|  | macrophage fate specification |
|  | macrophage dedifferentiation |
|  | macrophage contact |
|  | macrophage structure |
|  | macrophage elongation |
|  | macrophage proliferative response |
|  | macrophage tethering |
|  | macrophage diapedesis |
|  | macrophage disruption |
|  | macrophage rolling |
|  | macrophage transepithelial migration |
|  | macrophage agglutination |
|  | macrophage selection |
|  | macrophage morphogenesis |
|  | macrophage volume |
|  | macrophage lifespan |
|  | macrophage compartmentalization |
|  | macrophage architecture |

---

|  |  |
| --- | --- |
| Pathway | CD Markers -> Expression Targets in Lymphoid System and Blood |
|  | GPCRs Family -> Expression Targets in Lymphoid System and Blood |
|  | Immunoglobulin Superfamily -> Expression Targets in Lymphoid System and Blood |
|  | PPARG expressed in cells assotiated with psoriasis |
|  | Neighbors of macrophage |
|  | 1\_Low-density lipoprotein internalization and the accumulation of lipids in atherosclerosis |
|  | 4\_2\_Insulin resistance: inflammation-related insulin resistance |
|  | 4\_3\_Insulin resistance: FFA-related insulin resistance |
|  | 1\_SERPINA1-associated pulmonary emphysema |
|  | 3\_Islet beta-cell destruction in T1D |
|  | 1\_cd\_Amyloid-beta in Alzheimer's disease |

---

|  |  |
| --- | --- |
| MedScan ID | 10000094 |

---

|  |  |
| --- | --- |
| Alias | macrophagocytic cells |
|  | immune macrophage |
|  | macrophage |
|  | macrophagocytic cell |
|  | macrophagic |
|  | clasmatocytes |
|  | macrophages |
|  | immune macrophages |
|  | monocyte derived macrophage |
|  | macrophage cell |
|  | mononuclear macrophage |
|  | macrophage cells |
|  | mononuclear macrophages |
|  | macrophagal |
|  | monocyte derived macrophages |
|  | macrophageal |
|  | clasmatocyte |

---

|  |  |
| --- | --- |
| NCIm ID | C1550652 |

---
